# The Impacts of the Presence of an Unfamiliar Dog on Emerging Adults’ Physiological and Behavioral Responses Following Social Exclusion

**DOI:** 10.3390/bs10120191

**Published:** 2020-12-14

**Authors:** Ilona Papousek, Katharina Reiter-Scheidl, Helmut K. Lackner, Elisabeth M. Weiss, Corinna M. Perchtold-Stefan, Nilüfer Aydin

**Affiliations:** 1 Biological Psychology Unit, Institute of Psychology, University of Graz, 8010 Graz, Austria; katharina.scheidl@edu.uni-graz.at (K.R.-S.); corinna.perchtold@uni-graz.at (C.M.P.-S.); 2 Otto Loewi Research Center, Division of Physiology, Medical University of Graz, 8010 Graz, Austria; helmut.lackner@medunigraz.at; 3 Clinical Psychology Unit, Institute of Psychology, University of Innsbruck, 6020 Innsbruck, Austria; Elisabeth.Weiss@uibk.ac.at; 4 Social Psychology Unit, Institute of Psychology, University of Klagenfurt, 9020 Klagenfurt, Austria; niluefer.aydin@aau.at

**Keywords:** social exclusion, companion dog, affiliation, transient heart rate response

## Abstract

Research indicates that non-human attachment figures may mitigate the negative consequences of social exclusion. In the current experiment, we examined how the presence of an unfamiliar companion dog in the laboratory effects physiological and behavioral reactions in female emerging adults after social exclusion compared to inclusion. Results revealed the beneficial effects of the dog: Socially excluded participants in the company of a dog showed less aggressive behavior in response to the hot sauce paradigm compared to excluded participants in the control condition. Furthermore, cardiac responses indicated mitigated perception of threat in a subsequent insult episode when a dog was present. The presence of a dog did not impact the most instantaneous, “reflexive” response to the social exclusion as revealed by characteristic cardiac changes. Together, the findings indicate that the presence of a companion dog takes effect in a later, reflective period following a social exclusion experience, which implicates relevant social elaboration and appraisal processes.

## 1. Introduction

The longing for social ties is a fundamental part of human nature [1]. Thus, feeling left out has powerful and immediate emotional consequences, ranging from mood deterioration to suicidal thoughts [1,2,3,4,5,6,7,8]. It is even more important that the experience of social exclusion elicits a variety of behavioral responses including prosocial, avoidant, as well as antisocial and aggressive behaviors [6,9,10,11,12,13,14,15,16,17,18,19,20,21]. 

Aside from characteristics of the individual, several situational variables may help to explain under which circumstances people respond pro- or antisocially to social exclusion [4,6,16,22,23,24,25,26,27]. Most important to the present study, more prosocial reactions were observed when socially excluded individuals believed that there was still a chance for regaining acceptance from their social environment. Individuals utilize various means in order to regain a sense of belonging after social threat. For example, thinking about one’s own social identity [28], anticipating inclusion by other individuals [12,29], and the use of para-social attachment figures as social surrogates [30,31,32] all reduced negative emotions and antisocial tendencies after social exclusion. Aydin et al. [33] extended these findings and demonstrated that the mere presence of an unfamiliar companion dog served as a source of social connection and helped socially excluded individuals to restore their threatened needs. While these findings are intuitive and impressive, little is known about how the presence of a dog effects behavioral responses after social exclusion.

Research on human–animal relationships and interactions has primarily used attachment theory or social support theory as the overarching theoretical context to explain beneficial socio-emotional effects of human–animal interactions [34]. As many people seem to experience relationships with companion animals similarly to relationships with humans, attachment-related processes may also apply to human–animal relationships [34]. However, as attachment takes some time to develop, it seems likely that processes related to attachment are primarily involved in enduring relationships with one’s own pet. Short-term restoration of acutely threatened social needs through an incidental encounter with an unfamiliar domestic animal (as in Aydin et al. [33]) may rather be integrated in social support theory. Due to its unconditional and non-judgmental nature [35], the perception of social support provided by a dog may be particularly effective. The empirical findings of Aydin et al. [33] supported the view that these feelings of non-judgmental social acceptance may be a crucial factor in the improved coping with social exclusion. However, to date, not much relevant research has been concerned with the incidental presence of an unfamiliar dog. Aside from the overarching theoretical context, little is known about how the presence of a dog impacts the psychological processes in the immediate vicinity of a social exclusion experience. The present study aims at such a more fine-grained understanding.

In his influential Temporal Need-Threat model, Williams [7] suggested that three sequential stages exist in the reactions after ostracism—a “reflexive stage” occurring immediately after social exclusion, a “reflective stage” during which people consider coping reactions, and a “resignation stage”, which occurs in response to chronic exclusion. The first “reflexive stage” triggers immediate feelings of pain, negative emotions, and leads to decreased satisfaction of fundamental needs. In this stage, conscious and controlled appraisal and regulation efforts have not yet begun to operate [7]. The instantaneous, reflexive response to the social threat is difficult to access by self-report (which always involves reflection), but it can be properly observed by means of the immediate transient heart rate response to the social exclusion cue [36]. The potential of using this specific physiological response, which can provide objective information on an individual’s most immediate and automatic response to a social experience, has not yet been utilized in the present context. Generally, no studies on the effect of the presence of a domestic animal on the instantaneous, “reflexive” response to social threat cues are available to date.

The size of the characteristic cardiac response, which occurs on the scale of a few heartbeats, depends on the severity of the experience of social threat, with considerable and meaningful variability among individuals [36,37,38,39,40,41,42,43]. Part of the variability in these instantaneous responses to social threat cues is explained by situational moderators. A robust finding is that cardiac responses to social threat cues were less pronounced when the rejection was expected due to prior negative experiences than when it was unexpected [37,38,39,41,42,43]. Moreover, attenuated cardiac responses to negative social evaluation were observed when subjects were not interested in a relationship with the perpetrator [43,44].

The observable pro- or antisocial behaviors after social exclusion are posited to be part of the second stage, the “reflective stage”. In this stage, people quickly begin to assess the situation and attempt to recover their threatened psychological needs [45]. According to Williams [7], this is the only stage where situational factors can impact the nature of behavioral outcomes (but see [46]).

In the current research, we set up an experiment in which one group of participants received a social exclusion message and one group received an inclusion message, closely following a procedure that was successfully employed in previous studies [36,44]. Half of the participants in each group were accompanied by a dog, who was trained to remain quiet in his place during the entire experiment [33]. To ensure that the dog’s effects could be attributed to more than just distraction, an indoor water fountain was used as the control. After the social exclusion/inclusion incidence, participants were requested to allocate hot sauce to the putative excluder or includer to measure their behavioral tendencies. Subsequently, their response to an additional offending, negative social-evaluative statement was recorded [38]. Emerging adulthood is often a socially challenging developmental period, characterized by social instability and recalibration of one’s conception of self and identity and the relationships to others [47,48,49,50]. This makes emerging adults vulnerable for social stressors such as social exclusion and puts them at risk for internalizing as well as externalizing symptoms. Effective coping with social stressors is particularly important in that period [51]. Thus, the present study was focused on emerging adults, a population that is relatively understudied in this study’s particular context.

Taken together, the aims of the present study were to investigate the potential impact of the (inactive) presence of a friendly dog on (1) the instantaneous, “reflexive” response to social exclusion in terms of the immediate transient heart rate response; on (2) actual exclusion-related behavior in terms of hot sauce allocated to the putative excluder, assumedly influenced by “reflective” processes following the social exclusion episode; on (3) the perception of threat in a subsequent insult episode, operationalized by the transient cardiac response to a deprecating social-evaluative statement by the former excluder or includer.

As no relevant indications were available from the literature, we had no specific hypothesis as regards the animal’s impact on the instantaneous, “reflexive” response to the initial social exclusion/inclusion message. On the basis of the previous findings of Aydin et al. [33], who demonstrated beneficial effects of the presence of a dog on subjectively reported indicators of distress (i.e., conscious, reflected distress) after an exclusion episode, we expected that the presence of a dog would influence the elaborative, reflective processes following the social exclusion experience. Consequently, it was hypothesized that the heightened tendency toward allocating more hot sauce to excluders compared to includers would be mitigated in participants sharing the company of the dog. In addition, if the presence of a dog influences reflective processes after social exclusion or inclusion, it may also moderate the response to a subsequent insult [52]. We generally expected the response to the deprecating social-evaluative statement to be more pronounced after prior inclusion than exclusion, due to greater unexpectedness [37,38,39,41,42,43,44]. The presence of a dog was expected to mitigate this effect and reduce the impact of the insult.

## 2. Materials and Methods

### 2.1. Participants

The sample comprised a total of *n* = 84 female university students of various faculties (mean age *M* = 21.1, *SD* = 2.4), who were pseudo-randomly assigned to the four experimental conditions (2 × 2 design: exclusion/presence of dog, exclusion/control stimulus, inclusion/presence of dog, inclusion/control stimulus; including 22, 21, 20, and 21 participants, respectively), using the participants’ serial numbers and a predefined schedule. The choice of sample size was based on previous work demonstrating effects of an animal on the impact of social exclusion [33], effects of experimentally manipulated situational factors on antisocial behavior following exclusion in the hot sauce paradigm [27], and differences in immediate cardiac responses to social rejection versus acceptance and negative social evaluation [36,40,44]. The sample size is in the range of those previous studies or exceeds it, suggesting that the present sample size would provide adequate statistical power. Inclusion criteria were: age 18–30, female gender. Individuals were not included in the study if they reported major psychiatric disorders or a history of major psychiatric disorders according to the Structured Clinical Interview for DSM-IV Axis I Disorders (SCID-I; 3 candidates not included), use of psychoactive or cardioactive medication (one candidate not included), and allergy to or fear of dogs (no cases). Among the participants in the company of the dog during the experiment, *n* = 11 (exclusion group) and *n* = 9 (inclusion group) owned a pet (χ^2^_1, *n* = 42_ = 0.11, *p* = 0.746). Of those, *n* = 4 participants (exclusion group) and *n* = 3 (inclusion group) owned a dog. A female sample was chosen because their typical social structure may make women more vulnerable to social exclusion [53]; thus, we expected greater effect sizes in a female-only sample. All participants were Caucasian. Participants were recruited by emails sent out by the administration of the University of Graz and an announcement distributed by the Biological Psychology department via Facebook. Interested students sent their phone number to the experimenter and were called to fix an appointment. Participants were invited to a study on physiological responses to food additives. At the beginning of the experiment, they were informed that they were assigned to the capsaicin-group, in order to back up the use of hot chili sauce later in the experiment. Participation was on a voluntary basis. The study was conducted in accordance with the Declaration of Helsinki, and the protocol was approved by the ethics committee of the University of Graz (approval date 27 March 2015; code GZ.39/40/63 ex 2014/15). Written consent was obtained from all participants.

### 2.2. Experimental Manipulation Protocol

A realistic and validated protocol was used for the exclusion/inclusion manipulation, in which participants were excluded (versus included) by an ostensible group of colleagues on basis of their personal traits and preferences [36,44,54]; these studies also provided evidence for the validity of this protocol in terms of inducing the experience of exclusion. As a cover story, participants were told that a group of other students were waiting in an adjacent room for a brief discussion whether capsaicin should be mentioned as a food additive on groceries packages. The discussion would take place after the recording of their physiological response to capsaicin. First, participants were asked to complete a short questionnaire about personal preferences and interests such as their favorite music style and sitcom, their eating habits, and favorite tastes as well as character traits such as “being spontaneous”. After they had completed the questionnaire, participants were told that subject to their approval, the waiting discussion group should get the opportunity to study the answers on these questions, in order to become acquainted with the new group member while waiting. All participants agreed. By use of a pretended intercom, the experimenter announced the delivery to the alleged waiting students in the adjacent room: “I am coming”. Via the intercom, simulated by a pre-recorded message played back on a computer, the allegedly waiting other participants confirmed: “Alright!”. The experimenter took the completed questionnaire and left the room for two minutes and returned. While she explained the upcoming procedure in detail, the exclusion or inclusion statement was delivered via the fake intercom (1 min after the experimenter had returned). At the beginning of the unexpected utterance, the experimenter stopped talking and pretended to check the intercom.

Standardized (pre-recorded) statements were used. Exclusion statements were composed of the phrases: “Now, we could really do without this girl!”, “Yes, that’s really true” (translated from German). Inclusion statements were composed of the phrases: “She perfectly fits in our group!”, “Yes, that’s really true”. To exclude possible effects of the excluding/including persons’ gender, all statements were spoken by a male and a female voice. Half of the participants received the statement with a male voice speaking the excluding/including phrase and a female voice agreeing, in the other half this order was reversed. Sound levels of the statements were matched (65 dB). The statements were delivered at pre-determined times, controlled by the computer. Each statement lasted for about 5 s and started and ended with the clicking noise of activating the intercom, in order to make the participants believe that they overheard the utterances accidentally (sounding as if the intercom would have been activated by accident). Twenty seconds after the exclusion or inclusion statement, the experimenter left the room with the comment: “Oh, I am sorry, apparently someone has accidentally activated the intercom. I go and check it in the other room.” [36,44]. The experimenter returned after two minutes to start the hot sauce protocol.

In half of the experiments, a well-trained medium-sized domestic dog (Golden Retriever) was present. He was placed in a corner of the examination room where he remained quiet during the whole procedure. The dog was introduced to the participant by his name and his presence was explained by him being the experimenter’s faithful animal companion. At the end of the experiment all participants were asked and confirmed that the dog did not approach them while the experimenter was outside the room. The dog was a recognized assistance dog. It was trained and used to execute commands such as to stay lying down for at least 15 min. Thus, the test sessions matched a typical training situation for the dog. The dog owner was always present and took care of the welfare of his dog. During the test sessions he stayed next door to the examination room. After each test session, the dog was given a break and received a treat from its owner. No more than four experimental sessions were scheduled on one day, and there were no more than two experimental days in a week. In the control group, an indoor water fountain replaced the dog, in order to ensure that the dog’s effects can be attributed to more than just distraction. While research indicated that even inanimate objects such as blankets can provide some psychological comfort to distressed individuals, this applied only if these objects were familiar substitutional attachment agents or anthropomorphized to some degree. No such effects were observed when objects were functionally meaningless to the individual [55,56]. Therefore, while serving the purpose of holding the degree of attracted attention toward the stimulus and thus keeping the level of distraction constant across conditions, the water fountain was clearly not qualified for being perceived as a social surrogate to restore social connection.

### 2.3. Hot Sauce Protocol

The rationale of the well-validated hot sauce protocol is that participants are asked to allocate hot sauce to another person knowing that this person does not like spicy food [57], which is classified as an active and indirect form of aggressive behavior [58,59]. A big can of chili sauce (Marc’s Hot Chili Dippingsauce^TM^), which had been weighted before using a precision scale, was placed on the table. Participants were asked to taste and rate the spiciness of the sauce (1 “only a little hot” to 7 “extremely hot”), before allocating a self-decided amount of sauce to themselves and to a person out of the group from whom they believed having been excluded or included before. The participant was told that there was only this one can of sauce and, therefore, the person from the discussion group was not able to do it on his or her own. Participants were informed that the other person does not like spicy food, but that they nevertheless should not hesitate to determine the amount of sauce at their own discretion. They also got the information that the participant herself as well as the other person would be required to eat the whole amount of allocated sauce, in order to properly measure the physiological response to its consumption. The participants spooned the sauce into opaque cups [60]. After explaining the procedure, the experimenter left the room saying: “I will wait outside until you have finished your task without rushing. Please call me when you are done”. The experimenter returned after approximately two minutes and claimed to bring the cup for the other person in the nearby room. Outside the cup was weighted. The median of the spiciness ratings was *Md* = 4 (*IQR* = 2).

### 2.4. Recording of Cardiac Responses

The electrocardiogram (ECG) was recorded continuously using a portable device (eMotion Faros 180^TM^; sampling rate 1000 Hz). Disposable electrodes were placed at the thoracic region (2-lead, 1 channel position). R-waves were detected using a revised MATLAB function (MATLAB^TM^, MathWorks, Natick, MA, USA) based on the BioSig toolbox (http://biosig.sf.net), and the instantaneous (beat-to-beat) heart rate was calculated. In order to obtain highly synchronized data related to the stimuli, the beat-to-beat values were resampled at 4 Hz, using piecewise cubic spline interpolation after semiautomatic artifact correction, to obtain heart rate time series with equidistant time steps. Heart rate time series from five seconds preceding the exclusion/inclusion or further offence stimulus to 20 s after stimulus onset, i.e., exclusion/inclusion stimulus, [36,40] or 45 s after stimulus onset, i.e., later insult stimulus, [44] were segmented in 5 s intervals, thereby largely excluding influences of respiration (modulation of heart rate by respiratory sinus arrhythmia) [36,44]. The more extended time-window following the later insult stimulus was chosen, because previous research pointed to the potential significance of heart rate changes beyond 20 s [44]; the experiment’s time schedule did not allow the analysis of a more extended time window after the exclusion/inclusion stimulus). Averaged heart rates (in beats per minute, bpm) of the 5 s intervals were used in the statistical data analysis.

The experience of sudden social threat is followed by immediate transient heart rate deceleration that exceeds a typical orienting response [36,37,38,39,40,41,42,43,44]. This is in line with the characteristic association of fear elicitors implicating a high degree of self-involvement and imminence of threat with heart deceleration (“fear bradycardia”) [61,62,63,64,65]. According to traditional cognitive interpretations of transient heart rate deceleration [66,67], it implicates prolonged enhanced sensory intake and attention [38,39].

### 2.5. Procedure

After completion of the short questionnaire on personal traits and preferences, ECG electrodes were attached, and the participants were asked to remain seated. Then, the exclusion/inclusion manipulation and the hot sauce protocol were carried out. Forty seconds after the experimenter had left to allegedly deliver the cup of chili sauce to the other room, a pre-recorded negative social-evaluative, insulting statement was delivered via the fake intercom: “Why does this take so long? Is it that hard to spoon a little chili sauce into a cup?”. In half of the sample, this question was spoken by a female voice, in half of the sample by a male voice. Fifty seconds later, the experimenter returned and asked the participant to eat her own sauce. Then, the participants were informed that the planned discussion could not take place [36]. Participants were fully informed about the research questions and findings of the study via email after completion of the study. 

### 2.6. Statistical Analysis

The potential impact of the presence of a dog on the instantaneous, “reflexive” response to social exclusion was analyzed using heart rate as the dependent variable, in a 2 (exclusion vs. inclusion message; between-subjects factor) × 2 (presence of dog vs. control stimulus; between-subjects factor) × 5 (time: five 5-s periods starting from 5 s preceding the exclusion/inclusion message; within-subjects factor) analysis of variance. To analyze the impact of the presence of a dog on actual exclusion-related behavior, assumedly influenced by “reflective” processes following the social exclusion episode, a 2 (prior exclusion vs. inclusion; between-subjects factor) × 2 (presence of dog vs. control stimulus; between-subjects factor) analysis of variance was conducted using the amount of chili sauce assigned to the alleged other person as the dependent variable. The impact of the presence of a dog on the perception of threat in an additional insult episode was tested in a 2 (prior exclusion vs. inclusion; between-subjects factor) × 2 (presence of dog vs. control stimulus; between-subjects factor) × 10 (time: the 5-s periods starting from 5 s preceding the negative social-evaluative statement by the former excluder or includer) analysis of variance using heart rate as the dependent variable. As the four experimental groups were not fully homogenous regarding the mean age of participants (*F*(1,80) = 5.9, *p* = 0.018), and antisocial behavior decreases with age [68], age was entered as a covariate in these analyses. Supplemental analyses tested if there were mean differences between experimental groups in the amounts of chili sauce participants assigned to themselves (2, exclusion vs. inclusion × 2, presence of dog vs. control stimulus analyses of variance), and if the amount of assigned chili sauce was correlated with individual differences in the perceived spiciness of the sauce (Spearman’s Rho). All analyses were conducted with the software IBM SPSS statistics 26.

## 3. Results

**Figure 1:**
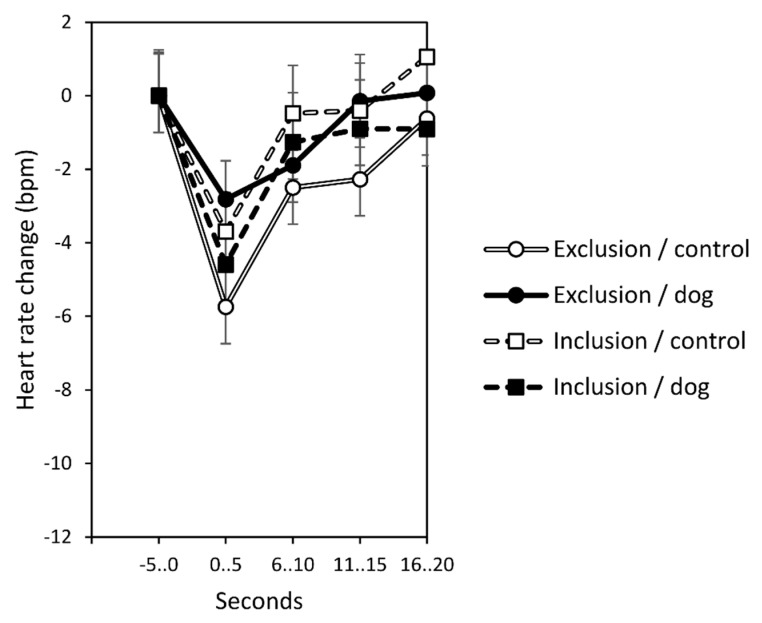
Immediate cardiac response to the exclusion/inclusion message. The offending statement occurred at second zero. Whiskers denote standard errors. The relevant interaction effect of exclusion/inclusion message by presence of dog/control stimulus by time is non-significant.

In the analysis of the instantaneous response to the exclusion/inclusion message, the relevant three-way interaction of time by exclusion/inclusion message by presence of dog/control stimulus was non-significant (*F*(4,316) = 0.8, *p* = 0.559; main effect time: *F*(4,316) = 3.2, *p* = 0.013, partial eta^2^ = 0.04; time × exclusion/inclusion: *F*(4,316) = 0.1, *p* = 0.973; time × dog/control: *F*(4,316) = 1.3, *p* = 0.261). From this, it follows that the presence of a dog did not moderate the typical time course of the transient cardiac response immediately after the experience of social exclusion (“reflexive” part of the response; Figure 1). 

**Figure 2:**
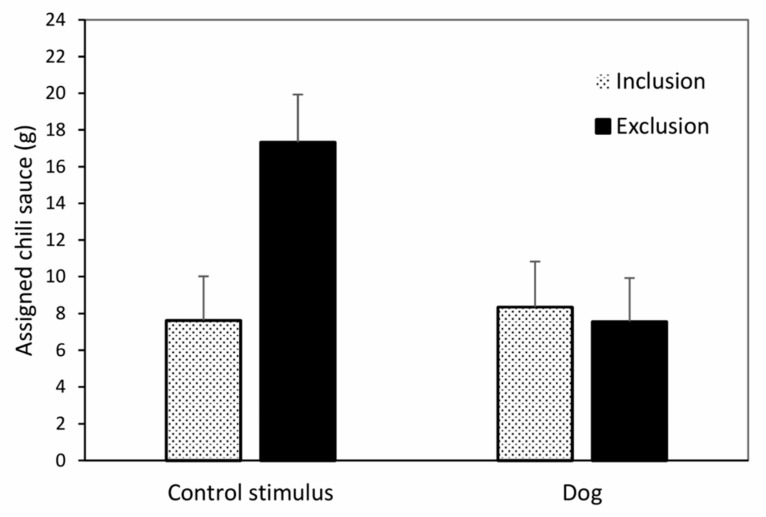
Impact of presence of a dog on exclusion-related antisocial behavior. Amount of hot sauce assigned to the putative perpetrator following social exclusion or inclusion. Significant interaction effect of prior exclusion/inclusion by presence of dog/control stimulus. Whiskers denote standard errors.

The analysis of exclusion-related behavior in terms of hot sauce allocated to the putative excluder showed the expected two-way interaction of prior exclusion/inclusion by presence of dog/control stimulus (*F*(1,79) = 4.5, *p* = 0.038, partial eta^2^ = 0.05); main effect exclusion/inclusion: *F*(1,79) = 3.3, *p* = 0.075; main effect dog/control: *F*(1,79) = 3.2, *p* = 0.077). Mean amounts of assigned chili sauce in the four experimental groups are illustrated in Figure 2. The expected heightened antisocial behavior following social exclusion (compared to inclusion) in terms of greater amounts of hot sauce assigned to a putative excluder (than to an includer) was only observed when the dog was not present (*t*(39) = 2.3, *p* = 0.030, eta^2^ = 0.12; control: *t*(39) = 0.4, *p* = 0.729, eta^2^ < 0.01).

**Figure 3:**
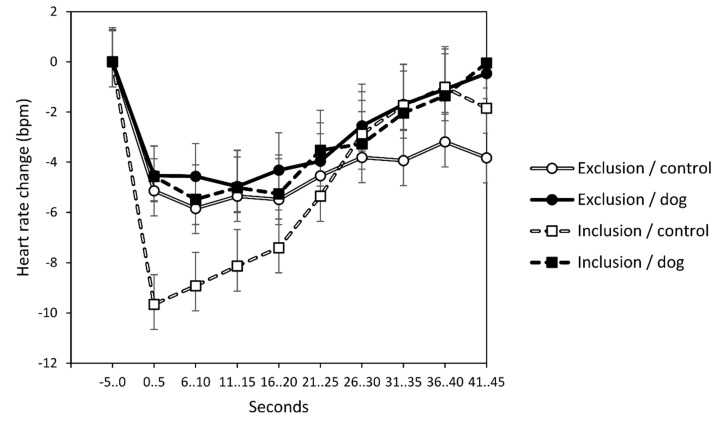
Immediate cardiac response to later insulting social-evaluative statement. Significant interaction effect of prior exclusion/inclusion by presence of dog/control stimulus by time. The essential difference is in the early part of the response (seconds 0–15). The offending statement occurred at second zero. Whiskers denote standard errors.

In the analysis targeting differences in the immediate perception of threat upon an additional insult, the expected three-way interaction of time by prior exclusion/inclusion by presence of dog/control stimulus was significant (*F*(9711) = 2.1, *p* = 0.030, partial eta^2^ = 0.03; main effect time: *F*(9711) = 1.0, *p* = 0.434; time x prior exclusion/inclusion: *F*(9711) = 2.5, *p* = 0.009, partial eta^2^ = 0.03; time × dog/control: *F*(9711) = 1.3, *p* = 0.258). The relevant three-way interaction is illustrated in Figure 3. It shows that the enhancing effect of greater unexpectedness of the negative social-evaluative statement (due to prior inclusion) on the decelerative cardiac response (indicating perception of social threat) was only present when participants did not have company of the dog. In the later course of the responses (from second 31 onwards), it seems that the response was prolonged in the group unaccompanied by a dog and prior exclusion compared to the other groups. This could not be secured statistically, however. Comparison of nadir (seconds 0–5) between prior exclusion and inclusion conditions within the participants not accompanied by the dog resulted in: *t*(39) = 2.7, *p* = 0.011; seconds 6–10: *t*(39) = 2.5, *p* = 0.018; seconds 11–15: *t*(39) = 2.5, *p* = 0.016; seconds 16–20: *t*(39) = 2.2, *p* = 0.033; seconds 21–25: *t*(39) = 1.8, *p* = 0.074; further time frames: all *p* ≥ 0.200. Single comparisons within participants accompanied by the dog were all *p* > 0.700.

The experience of social exclusion versus inclusion did not significantly influence how much hot sauce the participants assigned to themselves (main effect exclusion/inclusion: *F*(1,79) = 2.4, *p* = 0.122), and the presence of the dog did not moderate this relationship (interaction exclusion/inclusion × dog/control: *F*(1,79) = 1.2, *p* = 0.274). The correlations between perceived spiciness of the sauce (subjective rating) and the amount of chili sauce assigned to the putative other person was *r* = −0.08, *p* = 0.500. The correlation between the rating and the amount of sauce assigned to oneself was *r* = −0.21, *p* = 0.057.

## 4. Discussion

The present research replicates and extends previous findings of Aydin et al. [33] who demonstrated beneficial effects of the presence of a dog on self-reported (i.e., reflected) indicators of distress following social exclusion. In the current study, it was shown that the mere presence of an unfamiliar companion dog can impact objective physiological and behavioral outcomes after social exclusion by providing a source of social succor. In particular, the findings suggest that the presence of a dog primarily takes effect in a later, reflective period following a social exclusion experience, which implicates elaboration and appraisal of the exclusion episode. The presence of a dog does not seem to impact an individual’s most immediate, “reflexive” response to a social exclusion cue.

In line with previous research supporting the exclusion–aggression hypothesis [6,18,69], more antisocial behavior in terms of spiteful treatment of another person was observed after the experience of social exclusion compared to inclusion. However, congruent with our hypothesis, this effect was abolished when participants were in the company of a dog. This finding can be explained by Williams’ [7] theorizing the need for fortification in the reflective stage: socially excluded participants who were in the company of a dog could fortify their basic belonging needs. In the initial study of Aydin et al. [33], empirical evidence for the notion was provided in that the dog’s effect was mediated by enhanced feelings of non-judgmental social acceptance. Thus, we assume that in the present study, the dog may have mitigated the belonging threat experience by providing surrogate social connection, which then also attenuated reactive aggressive tendencies. 

These social elaborative processes stimulated by the presence of the dog also influenced the participants’ response to a later insult. The shape of the cardiac response (Figure 3) to the deprecating statement corresponded to the typical response following the experience of insult [40,70]. The sudden heart rate slowing indicates imminence of threat (“fear bradycardia”) and is related to heightened attention toward the source of threat and readiness for the intake of information [38,39,40,61,62,63,64,65,66,67]. Participants who had experienced social acceptance in the first place and did not have company of a dog clearly showed the most pronounced response to the insult. Thus, it seemed that in line with previous research, unexpectedness of the insult, brought about by prior experience of social acceptance by the same person, augmented the immediate threat response to the deprecating statement [37,38,39,41,42,44]. At the same time, the presence of the dog apparently mitigated this amplifying effect of the unexpectedness of the social threat.

Our primary explanation for this effect is that the presence of a dog may strengthen the feeling of social acceptance, thereby making subsequent acceptance from a (more distant) human less important. By making the social relationship with the perpetrator of the insult less important, the presence of the dog may have reduced the participant’s interest in the relationship with that person and, consequently, attenuated the immediate threat response to the insult. This interpretation is in accordance with previous research demonstrating that less interest in a relationship with the perpetrator attenuated the cardiac threat response to negative social evaluation [43,70]. 

The presence of a dog did not impact the instantaneous, “reflexive” part of the response to social exclusion. This observation is in line with research in this area suggesting that reflexive reactions are resistant to moderation by situational factors [71]. This “reflexive” component has been compared to the experience of pain, which triggers cautious attention and more elaborate cognitive processes in service of the appraisal of the meaning and importance of the potential threat [72]. Therefore, one of the central findings of the present study is that beneficial processes stimulated by the presence of a dog only take effect after some elaboration and reflection.

In this study, our focus was on objective and well-selected validated behavioral and physiological outcome variables instead of relying on subjective ratings of experiences, which is relatively uncommon in social psychologically oriented research designs. This approach, which was inspired by the interdisciplinary composition of the research team, has several advantages. Most importantly, self-report naturally involves reflection. Thus, the reflexive response, which per definition occurs at a stage in which conscious appraisal processes (i.e., reflection) have not yet begun to operate [7], is difficult to access by self-report. Further, participants are often not aware of their feelings or motivations that affect their behavior and physiology [73]. Behavioral and physiological variables do not require participants’ willingness to accurately report on their experiences, which may be constrained by self-presentational concerns or cultural norms [74]; and they are not influenced by conscious or unconscious regulatory control and other influences hampering the accurateness of self-reports [75,76]. Many laboratory stimuli do not provoke sufficiently strong and sustained emotional responses in order to be reflected in retrospective self-reports, but even subtle and fleeting experiences manifest in transitory physiological changes, and they can be measured without interrupting the process flow [75,77].

Notwithstanding these advantages of physiological and behavioral indicators, future studies may consider including at least some subjective judgments, too. On the other hand, future studies may additionally involve physiological data on the dog. Not only is the welfare of animals an increasingly recognized issue in the field of animal-assisted therapy [78,79,80]; part of the supportive and soothing effects of companion dogs may be explained by physiological synchronization with the animal, similar to its occurrence in human–human interactions [81,82]. An undisputed limitation of the study is that it does not allow a conclusion on whether there is something special to the presence of a dog. Similar effects may be produced by other pets, or any living being for that matter. Future studies may address this more in-depth question. Another important issue for further research will be the integration of the present and similar research findings in an overarching theoretical context such as social support theory. A further important limitation is the homogeneous study sample, which was exclusively Caucasian, female, and drawn from a university population, and thus limits the generalizability of findings. Especially men might in part experience social exclusion as less threatening than women and, thus, average effects might be smaller in men than in women [83,84]. Perhaps even more important, it should be noted that this research is culture-specific. It is very likely that the effects in the present study depend on the positive stereotype that is attached to friendly dogs in certain, but not all, cultures. Finally, a range of individual attitudes towards and experiences with dogs may moderate the reported effects.

Other research using less specific outcome variables than the present study suggested that beneficial effects of companion animals may also extend to other social situations. For instance, Polheber and Matchock [85] reported that the company of an unfamiliar dog mitigated the effects of an experimental condition implicating severe social evaluation stress on global physiological stress indicators. The effect of the dog even exceeded that of human friends in that study. Similar effects were observed in children who were exposed to difficult social situations with an unfamiliar pet or dog present [86,87]. Together, these and the present findings, which are not confined to pet owners, open up a particularly broad range of possible applications. They suggest that the mere presence of an unfamiliar dog may already be a fruitful add-on to psychotherapeutic interventions, not only in children but also in emerging adults, since social anxiety, insecure attachment, and excessive rejection sensitivity are frequent in this vulnerable developmental period and in many mental disorders.

## References

[1] Baumeister R.F., Leary M.R. (1995). The need to belong: Desire for interpersonal attachments as a fundamental human motivation. Psychol. Bull..

[2] Baumeister R.F., Tice D.M. (1990). Anxiety and social exclusion. J. Soc. Clin. Psychol..

[3] Chen Z., Poon K.-T., DeWall N., Jiang T. (2020). Life lacks meaning without acceptance: Ostracism triggers suicidal thoughts. J. Pers. Soc. Psychol..

[4] Gerber J., Wheeler L. (2009). On being rejected: A meta-analysis of experimental research on rejection. Perspect. Psychol. Sci..

[5] Leary M.R., Tambor E.S., Terdal S.J., Downs D.L. (1995). Self-esteem as an interpersonal monitor. The sociometer hypothesis. J. Pers. Soc. Psychol..

[6] Warburton W.A., Williams K.D., Cairns D.R. (2006). When ostracism leads to aggression: The moderating effects of control deprivation. J. Exp. Soc. Psychol..

[7] Williams K.D. (2009). Ostracism: A temporal need-threat model. Adv. Exp. Soc. Psychol..

[8] Zadro L., Williams K.D., Richardson R. (2004). How low can you go? Ostracism by a computer lowers belonging, control, self-esteem, and meaningful existence. J. Exp. Soc. Psychol..

[9] DeWall C.N., Baumeister R.F. (2006). Alone but feeling no pain. Effects of social exclusion on physical pain tolerance and pain threshold, affective forecasting, and interpersonal empathy. J. Pers. Soc. Psychol..

[10] DeWall C.N., Maner J.K., Rouby D.A. (2009). Social exclusion and early-stage interpersonal perception: Selective attention to signs of acceptance. J. Pers. Soc. Psychol..

[11] Hopkins Z.L., Branigan H.P. (2020). Children show selectively increased language imitation after experiencing ostracism. Dev. Psychol..

[12] Gardner W.L., Pickett C.L., Brewer M.B. (2000). Social exclusion and selective memory: How the need to belong influences memory for social events. Pers. Soc. Psychol. Bull..

[13] Kirkpatrick L.A., Waugh C.E., Valencia A., Webster G.D. (2002). The functional domain specificity of self-esteem and the differential prediction of aggression. J. Pers. Soc. Psychol..

[14] Lakin J.L., Chartrand T.L., Arkin R.M. (2008). Using nonconscious behavioural mimicry to create affiliation and rapport. I am too just like you: Nonconscious mimicry as an automatic behavioural response to social exclusion. Psychol. Sci..

[15] Pickett C.L., Gardner W.L., Knowles M. (2004). Getting a cue: The need to belong and enhanced sensitivity to social cues. Pers. Soc. Psychol. Bull..

[16] Richman L., Leary M.R. (2009). Reactions to discrimination, stigmatization, ostracism, and other forms of interpersonal rejection: A multimotive model. Psychol. Rev..

[17] Riva P., Williams K.D., Torstrick A.M., Montali L. (2014). Orders to shoot (a camera): Effect of ostracism on obedience. J. Soc. Psychol..

[18] Twenge J.M., Baumeister R.F., Tice D.M., Stucke T.S. (2001). If you can’t join them, beat them: Effects of social exclusion on aggressive behaviour. J. Pers. Soc. Psychol..

[19] Twenge J.M., Baumeister R.F., DeWall N.C., Ciarocco N.J., Bartels J.M. (2007). Social exclusion decreases prosocial behaviour. J. Pers. Soc. Psychol..

[20] Wesselmann E.D., Ren D., Williams K.D. (2015). Motivations for responses to ostracism. Front. Psychol..

[21] Williams K.D., Cheung C.K.T., Choi W. (2000). Cyberostracism: Effects of being ignored over the internet. J. Pers. Soc. Psychol..

[22] Aydin N., Krueger J., Frey D., Kastenmüller A., Fischer P. (2014). Social exclusion and xenophobia: Intolerant attitudes toward ethnic and religious minorities. Group Process. Intergroup Relat..

[23] Aydin N., Agthe M., Pfundmair M., Frey D., DeWall C.N. (2017). Safety in beauty. Social exclusion, antisocial responses, and the desire to reconnect. Soc. Psychol..

[24] DeWall C.N., Richman S.B. (2011). Social exclusion and the desire to reconnect. Soc. Pers. Psychol. Compass.

[25] Miao X.-Y., Chan K.Q., Gao C., Lv S.S., Zhu Y., Wang Z.-J. (2020). Underdogs make an alliance: The co-experience of rejection promotes cooperation. Brit. J. Soc. Psychol..

[26] Pillny M., Lincoln T.M. (2020). The demotivating effect of social exclusion: An experimental test of a psychosocial model on the development of negative symptoms in psychosis. Schizophr. Res..

[27] Wesselmann E.D., Butler F.A., Williams K.D., Pickett C.L. (2010). Adding injury to insult: Unexpected rejection leads to more aggressive responses. Aggress. Behav..

[28] Knowles M.L., Gardner W.L. (2008). Benefits of membership: The activation and amplification of group identities in response to social rejection. Pers. Soc. Psychol. Bull..

[29] Maner J.K., DeWall C.N., Baumeister R.F., Schaller M. (2007). Does social exclusion motivate interpersonal reconnection? Resolving the “porcupine problem”. J. Pers. Soc. Psychol..

[30] Aydin N., Fischer P., Frey D. (2010). Turning to god in the face of ostracism: Effects of social exclusion on religiousness. Pers. Soc. Psychol. Bull..

[31] Gardner W.L., Knowles M.L. (2008). Love makes you real: Favorite television characters are perceived as “real” in a social facilitation paradigm. Soc. Cogn..

[32] Hales A.H., Wesselmann E.D., Williams K.D. (2016). Prayer, self-affirmation, and distraction improve recovery from short-term ostracism. J. Exp. Soc. Psychol..

[33] Aydin N., Krueger J., Fischer J., Hahn D., Frey D., Kastenmüller A., Fischer P. (2012). “Man’s best friend:” How the presence of a dog reduces mental distress after social exclusion. J. Exp. Soc. Psychol..

[34] Meehan M., Massavelli B., Pachana N. (2017). Using attachment theory and social support theory to examine and measure pets as sources of social support and attachment figures. Anthrozoös.

[35] Pachana N.A., Massavelli B.M., Robleda-Gomez S., Blazina C., Boyraz G., Shen-Miller D.S. (2011). A developmental psychological perspective on the human-animal bond. The Psychology of the Human-Animal Bond: A Resource for Clinicians and Researchers.

[36] Reiter-Scheidl K., Papousek I., Lackner H.K., Paechter M., Weiss E.M., Aydin N. (2018). Aggressive behavior after social exclusion is linked with the spontaneous initiation of more action-oriented coping immediately following the exclusion episode. Physiol. Behav..

[37] Dekkers L.M.S., van der Molen M.J.W., Moor B.G., van der Veen F.M., van der Molen M.W. (2015). Cardiac and electro-cortical concomitants of social feedback processing in women. Soc. Cogn. Affect. Neurosci..

[38] Gunther Moor B.G., Crone E.A., van der Molen M.W. (2010). The heartbrake of social rejection: Heart rate deceleration in response to unexpected peer rejection. Psychol. Sci..

[39] Gunther Moor B.G., Bos M.G., Crone E.A., van der Molen M.W. (2014). Peer rejection cues induce cardiac slowing after transition into adolescence. Dev. Psychol..

[40] Papousek I., Aydin N., Lackner H.K., Weiss E.M., Bühner M., Schulter G., Charlesworth C., Freudenthaler H.H. (2014). Laughter as a social rejection cue: Gelotophobia and transient cardiac responses to other persons’ laughter and insult. Psychophysiology.

[41] Van der Veen F.M., van der Molen M.W., Sahibdin P.P., Franken I.H. (2014). The heart-brake of social rejection versus the brain wave of social acceptance. Soc. Cogn. Affect. Neurosci..

[42] Van der Veen F.M., van der Molen M.J., van der Molen M.W., Franken I.H. (2016). Thumbs up or thumbs down? Effects of neuroticism and depressive symptoms on psychophysiological responses to social evaluation in healthy subjects. Cogn. Affect. Behav. Neurosci..

[43] Van der Veen F.M., Burdzina A., Langeslag S.J.E. (2019). Don’t you want me, baby? Cardiac and electrocortical concomitants of romantic interest and rejection. Biol. Psychol..

[44] Lackner H., Reiter-Scheidl K., Aydin N., Perchtold C.M., Weiss E.M., Papousek I. (2018). Laughter as a social rejection cue: Influence of prior explicit experience of social rejection on cardiac signs of ‘freezing’. Int. J. Psychophysiol..

[45] Williams K.D., Hales A.H., Michels C., Rudert S.C., Greifeneder R., Williams K.D. (2019). Social ostracism as a factor motivating interest in extreme groups. Current Directions in Ostracism, Social Exclusion, and Rejection Research.

[46] Hartgerink C.H.J., van Beest I., Wicherts J.M., Williams K.D. (2015). The ordinal effects of ostracism: A meta-analysis of 120 cyberball studies. PLoS ONE.

[47] Arnett J.J. (2000). Emerging adulthood: A theory of development from the late teens through the twenties. Am. Psychol..

[48] Arnett J.J. (2007). Emerging adulthood: What is it, and what is it good for?. Child Dev. Perspect..

[49] Lapsley D., Woodbury R.D., Arnett J.J. (2016). Social cognitive development in emerging adulthood. The Oxford Handbook of Emerging Adulthood.

[50] Schwartz S.J., Syed M., Yip T., Knight G.P., Umana-Taylor A.J., Rivas-Drake D., Lee R.M. (2014). Methodological issues in ethnic and racial identity research with ethnic minority populations: Theoretical precision, measurement issues, and research designs. Child Dev..

[51] Holterman L.A., Murray-Close D.K., Breslend N.L. (2016). Relational victimization and depressive symptoms: The role of autonomic nervous system reactivity in emerging adults. Int. J. Psychophysiol..

[52] Liddell B.J., Courtney B.S. (2018). Attachment buffers the physiological impact of social exclusion. PLoS ONE.

[53] Benenson J.F., Markovits H., Hultgren B., Nguyen T., Bullock G., Wrangham R. (2013). Social exclusion: More important to human females than males. PLoS ONE.

[54] Pfundmair M., DeWall C.N., Fries V., Geiger B., Krämer T., Krug S., Frey D., Aydin N. (2015). Sugar or spice: Using I3 metatheory to understand how and why glucose reduces rejection-related aggression. Aggressive Behav..

[55] Pancani L., Riva P., Sacchi S. (2019). Connecting with a slot machine: Social exclusion and anthropomorphization increase gambling. J. Gambl. Stud..

[56] Ybarra G.J., Passman R., Eisenberg C.S. (2000). The presence of security blankets or mothers (or both) affects distress during pediatric examinations. J. Consult. Clin. Psychcol..

[57] Lieberman J.D., Solomon S., Greenberg J., McGregor H.A. (1999). A hot new way to measure aggression: Hot sauce allocation. Aggress. Behav..

[58] McCarthy R.J., Elson A. (2018). A conceptual review of lab-based aggression paradigms. Collab. Psychol..

[59] Parrott P.J., Giancola P.R. (2007). Addressing ‘The criterion problem’ in the assessment of aggressive behavior: Development of a new taxonomic system. Aggress. Violent. Behav..

[60] Ayduk Ö., Gyurak A., Luerssen A. (2008). Individual differences in the rejection-aggression link in the hot sauce paradigm: The case of rejection sensitivity. J. Exp. Soc. Psychol..

[61] Adenauer H., Catani C., Keil J., Aichinger H., Neuner F. (2010). Is freezing an adaptive reaction to threat? Evidence from heart rate reactivity to emotional pictures in victims of war and torture. Psychophysiology.

[62] Campbell B.A., Wood G., McBride T., Lang P.J., Simons R.F., Balaban M.T. (1997). Origins of orienting and defensive responses: An evolutionary perspective. Attention and Orienting: Sensory and Motivational Processes.

[63] Hagenaars M.A., Stins J.F., Roelofs K. (2012). Aversive life events enhance human freezing responses. J. Exp. Psychol. Gen..

[64] Kreibig S.D. (2010). Autonomic nervous system activity in emotion: A review. Biol. Psychol..

[65] Lang P.J., Wangelin B.C., Bradley M.M., Versace F., Davenport P.W., Costa V.D. (2011). Threat of suffocation and defensive reflex activation. Psychophysiology.

[66] Bradley M.M., Lang P.J., Cacioppo J.T., Berntson G. (2007). Emotion and motivation. Handbook of Psychophysiology.

[67] Graham F.K., Clifton R.K. (1966). Heart-rate change as a component of the orienting response. Psychol. Bull..

[68] Lehmann A., Ittel A. (2012). Aggressive behavior and measurement of psychopathy in female inmates of German prisons—A preliminary study. Int. J. Law Psychiatry.

[69] Leary M.R., Twenge J.M., Quinlivan E. (2006). Interpersonal rejection as a determinant of anger and aggression. Pers. Soc. Psychol. Rev..

[70] Vögele C., Sorg S., Studtmann M., Weber H. (2010). Cardiac autonomic regulation and anger coping in adolescents. Biol. Psychol..

[71] Wesselmann E.D., Williams K.D., DeWall C.N. (2013). Ostracism and stages of coping. Oxford Library of Psychology. The Oxford Handbook of Social Exclusion.

[72] Williams K.D. (2007). Ostracism. Annu. Rev. Psychol..

[73] Wright R.A., Kirby L.D. (2001). Cardiovascular response: An integrative analysis with applications in social psychology. Adv. Exp. Soc. Psychol..

[74] Egloff B., Wilhelm F.H., Neubauer D.H., Mauss I.B., Gross J.J. (2002). Implicit anxiety measure predicts cardiovascular reactivity to an evaluated speaking task. Emotion.

[75] Allen N.B., Kuppens P., Sheeber L.B. (2012). Heart rate responses to parental behavior in depressed adolescents. Biol. Psychol..

[76] Blascovich J., Seery M.D., Mugridge C.A., Norris K., Weisbuch M. (2004). Predicting athletic performance from cardiovascular indexes of challenge and threat. J. Exp. Soc. Psychol..

[77] Gerin W., Bovbjerg D.H., Glynn L., Davidson K., Sanders M., Sheffield D., Christenfeld N. (1999). Comment on negative emotions and acute cardiovascular responses to laboratory challenges. Ann. Behav. Med..

[78] Corsetti S., Ferrara M., Natoli E. (2019). Evaluating stress in dogs involved in animal-assisted interventions. Animals.

[79] Glenk L.M. (2017). Current perspectives on therapy dog welfare in animal-assisted interventions. Animals.

[80] Melco A.L., Goldman L., Fine A.H., Peralta J.M. (2020). Investigation of physiological and behavioral responses in dogs participating in animal-assisted therapy with children diagnosed with attention-deficit hyperactivity disorder. J. Appl. Anim. Welf. Sci..

[81] Lackner H., Feyaerts K., Rominger C., Oben B., Schwerdtfeger A., Papousek I. (2019). Impact of humor-related communication elements in natural dyadic interactions on interpersonal physiological synchrony. Psychophysiology.

[82] Palumbo R.V., Marraccini M.E., Weyandt L.L., Wilder-Smith O., McGee H.A., Liu S., Goodwin M.S. (2017). Interpersonal autonomic physiology: A systematic review of the literature. Pers. Soc. Psychol. Rev..

[83] Robinson J.L., Biringen Z. (1995). Gender and emerging autonomy in development. Psychoanal. Inq..

[84] Thibeault M.A., Stein G.L., Nelson-Gray R.O. (2018). Ethnic identity in context of ethnic discrimination: When does gender and other-group orientation increase risk for depressive symptoms for immigrant-origin young adults. Cult. Divers. Ethn. Min. Psychol..

[85] Polheber J.P., Matchock R.L. (2014). The presence of a dog attenuates cortisol and heart rate in the Trier Social Stress Test compared to human friends. J. Behav. Med..

[86] Beetz A., Julius H., Turner D., Kotrschal K. (2012). Effects of social support by a dog on stress modulation in male children with insecure attachment. Front. Psychol..

[87] Kertes D.A., Liu J., Hall N.J., Hadad N.A., Wynne C.D.L., Bhatt S.S. (2017). Effect of pet dogs on children’s perceived stress and cortisol stress response. Soc. Dev..

